# A Novel Nutritional, Thirst‐Free Drink Alleviates Type 2 Diabetes: From Toxicity Studies to Human Clinical Trials

**DOI:** 10.1155/bmri/8998340

**Published:** 2026-04-13

**Authors:** Tai-Chi Hung, Yue-Hua Deng, Yu-Hsin Hung, Win-Ping Deng, Pao-Chang Chiang, Chun-Hao Chan, Fang-Pey Chen, Hong-Jian Wei, Abhinay Kumar Singh, Chi-Sheng Chiou

**Affiliations:** ^1^ Stem Cell Research Center, College of Oral Medicine, Taipei Medical University, Taipei, Taiwan, tmu.edu.tw; ^2^ School of Dentistry, College of Oral Medicine, Taipei Medical University, Taipei, Taiwan, tmu.edu.tw; ^3^ Institute of Traditional Medicine, School of Medicine, National Yang Ming Chio Tung University, Taipei, Taiwan; ^4^ Dental Department, Wan Fang Hospital, Taipei Medical University, Taipei, Taiwan, tmu.edu.tw; ^5^ Center for Traditional Medicine, Taipei Veterans General Hospital, Taipei, Taiwan, nymu-e.web.ym.edu.tw; ^6^ Department of Radiation Oncology, Columbia University Irving Medical Center, New York, New York, USA, columbia.edu; ^7^ Division of Allergy, Immunology and Rheumatology, Department of Internal Medicine, School of Medicine, College of Medicine, Taipei Medical University, Taipei, Taiwan, tmu.edu.tw; ^8^ Division of Allergy, Immunology and Rheumatology, Department of Internal Medicine, Taipei Medical University Hospital, Taipei, Taiwan, tmuh.org.tw

**Keywords:** antidiabetic, herbal drink, T2DM, toxicity

## Abstract

**Background:**

Although extracts from medicine food homologous plants have been widely used as interventions for the prevention and treatment of Type 2 diabetes mellitus (T2DM), it remains a major public health concern. Diabetes is also known as a “thirsting disorder” because it leads to the depletion of vital biomolecules in the body regardless of food or water intake and interferes with the replenishment of fluids through drinking. We developed a novel thirst‐free drink (TF601) that contains a combination of traditional Chinese medicine food homologous plants extracts of *Psidium guajava*, *Momordica charantia,* Lycii Fructus, Glycyrrhizae Radix, and *Camellia sinensis.* In this study, we investigated TF601′s drink subchronic toxicity evaluation in mice and an exploratory, uncontrolled clinical assessment of TF601 in patients with T2DM.

**Methods:**

Herein, for toxicity studies, mice (male and female) were divided into a TF601‐treated group and a control group. The TF601 drink was orally administered for 90 days to evaluate its toxicity. The mice were monitored daily for the appearance of any clinical signs. Their food intake and body weight changes were measured every week. Hematological, biochemical, and histopathological changes were also evaluated. For the exploratory human study, 14 patients with T2DM were enrolled in an uncontrolled pilot trial and glucose levels, hemoglobin A1c percentage, oral glucose tolerance levels, and body weight were monitored from baseline to 2 months later.

**Results:**

Oral administration of TF601 drink in mice did not develop any clinical signs, nor did they die due to the treatment. No changes were noted in body weight, food intake, or liver, kidney, or brain histology. However, slightly significant changes were observed in the hematological and biochemical parameters only in male mice as compared with control mice. In the human clinical trial, significant improvements were noted in T2DM patients′ glucose levels, and liver and kidney functions after a 2‐month treatment with TF601. Overall, our toxicity studies in animals and clinical trials in humans revealed that TF601 drink was not toxic, alleviated the blood glucose level, and maintained body weight in T2DM patients.

**Conclusion:**

These findings suggest that TF601 is well tolerated in a 90‐day mouse toxicity study and may exhibit potential glucose‐modulating effects in patients with T2DM. However, the human data are preliminary, and controlled clinical trials with larger sample sizes are required to confirm efficacy and safety.

**Trial Registration:**

Center for Traditional Medicine of Taipei Veterans General Hospital, IRB of Taipei Veterans General Hospital, Taipei, Taiwan (2021‐08‐011C), on August 18, 2021 retrospectively registered. https://vghtpe.cims.tw/wiPtms/index.html.

## 1. Background

Most of the world′s population suffers from chronic diseases that are highly prevalent worldwide, in which the most common disease is Type 2 diabetes mellitus (T2DM) [1, 2]. Despite the availability of conventional pharmacotherapies, long‐term glycemic control remains challenging for many patients, prompting growing interest in complementary and dietary‐based interventions. Herbal plant extracts have been an integral part of traditional Chinese medicine since ancient times [3]. Medicinal plants are widely used for the discovery of newly formulated drugs. In recent years, there is an increasing awareness and use of traditional Chinese medicine, especially herbal preparations commonly called herbal medicine or drug [4]. Polyherbal preparations, in particular, are widely consumed as beverages or dietary supplements due to their perceived safety and multitarget therapeutic potential. Various traditional Chinese medicines are marketed without evaluating their safety and efficacy profiles. Owing to the lack of knowledge, traditional Chinese medicine prescriptions for comorbidities such as T2DM are limited, which necessitates investigations into the toxicity and clinical effect of these herbal drugs [5, 6]. Although plant‐based products are often assumed to be inherently safe, increasing evidence indicates that prolonged consumption, high‐dose exposure, or combined formulations may elicit adverse effects due to bioactive constituents, herb–herb interactions, or cumulative toxicity [7]. Therefore, rigorous preclinical safety assessment remains essential, even for herbal products categorized as functional foods.

Repeated‐dose oral toxicity studies are recommended as an initial step for safety profiling of newly formulated herbal products intended for long‐term consumption [8, 9]. Subchronic (90‐day) oral toxicity studies, in particular, provide valuable information regarding potential systemic toxicity, target organ effects, and sex‐related differences following prolonged exposure. In line with these regulatory principles, the present study evaluated the safety of a newly developed polyherbal thirst‐free drink, designated TF601, through a 90‐day repeated oral toxicity study in mice.

Extracts from herbal plants such as guava (*Psidium guajava*), bitter gourd (*Momordica charantia*), wolfberry (Lycii Fructus), licorice (Glycyrrhizae Radix), and green tea (*Camellia sinensis*) are widely used as drugs in traditional Chinese medicine to treat diseases, including cancer, diabetes, ulcer, hypertension, and neurological disorder [10–13]. The aforementioned plants possess antioxidative and antidiabetic properties [14]. Polyherbal extracts from these plants were combined (at a fixed ratio) to prepare a thirst‐free drink that is designated as TF601. Most herbal plants serve as food or food ingredients; these plants are also referred to as functional foods [15, 16].

Oral administration of *P. guajava* reduces fasting blood glucose (FBG) level and improves insulin sensitivity through the upregulation of *GLUT2* expression in hepatocytes [17]. Furthermore, *M. charantia* protects against T2DM through the modulation of the c‐Jun N‐terminal kinase and nuclear factor–*κ*B pathways [12]. Lycii Fructus, Glycyrrhizae Radix, and *C. sinensis* have also been demonstrated to exert antidiabetic effects through various mechanisms [18].

Despite extensive evidence supporting the biological activities of individual components, the safety and efficacy of their combined formulation as a polyherbal beverage remain insufficiently characterized. Importantly, long‐term consumption of multicomponent herbal formulations may produce unexpected toxicological effects that cannot be predicted from individual extracts alone [19]. Therefore, for their safety profiling, polyherbal drugs must be subjected to preclinical toxicity tests. In this study, we investigated TF601′s toxic effects in mice and its antidiabetic effects in patients with T2DM.

In this study, we performed a 90‐day repeated oral toxicity assessment of TF601 in male and female mice, focusing on clinical observations, body weight, hematological and biochemical parameters, and histopathological examination of major organs. In addition, an exploratory, uncontrolled pilot study was conducted in a small cohort of patients with T2DM to preliminarily assess glycemic outcomes and safety‐related clinical parameters. Together, these investigations are aimed at providing initial safety data and preliminary evidence supporting the potential use of TF601 as a functional polyherbal beverage for T2DM management, while clearly acknowledging the exploratory nature and limitations of the clinical findings.

## 2. Methods

### 2.1. TF601 Preparation

TF601 was derived from a commercially available beverage called Thirst‐free (Lisun Bio‐Medical Technology, Taipei, Taiwan). This herbal drink contains water and extracts from *P. guajava*, *M. charantia*, Lycii Fructus, Glycyrrhizae Radix, and *C. sinensis*. The raw materials of Thirst‐free were cooked under 100°C, filtered, hot‐filled, sterilized (120°C and 2.4 atm), and cooled before storage. Resistant dextrin was purchased from Union Food. TF601 was prepared by Der Kang Biology Technology. For freeze–drying under vacuum, Thirst‐free liquid was first mixed with resistant dextrin at a ratio of 6:1; subsequently, this mixture was dehydrated at −32°C and 0.05 PA by using a shelf freeze dryer. Then, it was stored at ≤ 30°C under 55% humidity.

### 2.2. Animal Studies

For toxicity studies, 6‐week‐old C57BL/6 mice were purchased from BioLAS Co., Taipei, Taiwan. All animal experiments were performed according to the Institutional Animal Care and Use Committee (IUCAC) of Taipei Medical University (Approval No. LAC‐2019‐0308). The animals were individually housed in cages (Type 3H, with aspen wood bedding) maintained under controlled, hygienic conditions (temperature: 21^°^C ± 2^°^C; hourly air change count: 10–15; relative humidity, 30%–70%; 12‐h light/dark cycle). The mice were acclimatized for 7 days before experiments. They received a rodent‐specific standard dry pellet diet ad libitum. All animal studies were conducted in accordance with the ARRIVE guidelines and those of Taipei Medical University′s Institutional Animal Care and Use Committee.

### 2.3. Toxicity Assessment

A total of 20 mice were used for toxicity studies. The highest dose of TF601 used for a subchronic study was 120 mg/kg body weight. The mice were randomly selected and divided into treatment and control groups. The mice in both groups were further stratified by sex into two subgroups: male and female groups. The study duration was 90 days. The polyherbal drug was orally administered (intragastric; dose: 120 mg/kg body weight/day) every week—Monday to Saturday—at a fixed time. The control groups were administered phosphate‐buffered saline.

### 2.4. Clinical Observations

The skin, fur, eyes, and mucous membranes of the mice were examined for potential clinical signs. In addition, we evaluated the drug′s effects on the animals′ respiratory system, circulatory system, autonomic nervous system (salivation), central nervous system (behavior), and motor system (tremor and convulsion, motor activity, gait and posture, and reactivity to handling and sensory stimuli).

### 2.5. Body Weight and Food Intake Measurement

To avoid causing stress in the animals, we measured group‐ and subgroup‐specific mean values. Total food intake per cage was monitored; weekly mean values calculated for each mouse′s food intake.

### 2.6. Hematological and Biochemical Analyses

Hematological parameters, such as red blood cell (RBC) count, hemoglobin A1c (HbA1c) percentage, white blood cell counts (numbers of neutrophils [NEU], lymphocytes [LYM], eosinophils, monocytes [MONO], and basophils), hematocrit (HCT)%, mean corpuscular hemoglobin (MCH), mean corpuscular volume (MCV), MCH concentration, mean platelet volume, and RBC distribution width‐coefficient of variation, were analyzed using XE2100 (Sysmex, Japan). Biochemical parameters, such as alanine aminotransferase (ALT), total protein, aspartate aminotransferase (AST), albumin, blood urea nitrogen (BUN), creatinine (CRE), triglyceride, and total cholesterol, were measured using the Cobas 8000 Biochemistry Automatic Analyzer (Roche, Germany).

### 2.7. Histopathological Analyses

No mouse died during the study period. At the end of the study, the mice were starved overnight (for 18 h) before euthanasia. The animals were subjected to deep anesthesia induced using isoflurane. Then, they were euthanized through CO_2_ exposure and exsanguinated through intracardiac injection in accordance with Directive 2010/63/EU. For histopathological analyses, liver, kidney, and brain tissues were harvested from the control and TF601‐treated (doses: 120 mg/kg body weight/day) mice. The obtained tissue samples were fixed in 10% buffered formalin for 24 h, processed following standard protocols, and embedded in paraffin wax. The samples were sectioned into 4‐*μ*m slices, which were stained with hematoxylin and eosin.

### 2.8. Clinical Trial

A pilot clinical trial was conducted at the Center for Traditional Medicine of Taipei Veterans General Hospital, Taiwan. The study protocol used was approved by the hospital′s institutional review board (Protocol Number 2021‐08‐011C). For the clinical trial, we screened 32 patients with T2DM who received treatment at our hospital and eventually enrolled 14 of them. This study included patients aged 20–75 years whose HbA1c percentages were 7%–10%, who received < 3 oral medicines for T2DM, and whose body mass index (BMI) was > 22 kg/m^2^. We excluded patients with other diabetes types, those who changed therapy or medicine during the study period, those who received insulin injection or any other Chinese medicine within 3 months before the present study, those who were pregnant or lactating, those with stressful conditions such as ketoacidosis, those with a serum ALT level of > 72 U/L or an estimated glomerular filtration rate (eGFR) of <60 mL/min/1.73 m^2^, and those with a history of cerebrovascular or cardiovascular disease. To protect patient privacy, codes were assigned to each included patient and these codes were used instead of their real names. The participants were instructed to fast overnight before visiting the hospital once a month. On each visit, an oral glucose tolerance test (OGTT) was conducted and blood samples were collected.

### 2.9. Measurement of Body Weight and FBG Levels

The participants regularly monitored (at home) their body weight (once a week) and FBG (thrice a week). For the self‐monitoring of FBG levels, each participant was provided with a glucometer (Chengxin Medical Equipment, Taipei, Taiwan, China), two packs of test chips (25 chips per pack; Chengxin Medical Equipment), and one pack of automatic blood lancets (50 lancets per pack; Chengxin Medical Equipment).

### 2.10. OGTT

For the OGTT, blood samples were collected at 0 and 120 min. Biochemical indicators of liver function (AST, ALT, and alkaline phosphatase [ALP]), kidney function (CRE and eGFR), and T2DM (HbA1c, FBG, fasting insulin, and 2‐h blood glucose [2hBG]) were evaluated. To prepare the glucose agent for OGTT, 50% glucose monohydrate (Sintong Biotech, Taiwan) was diluted with boiled water at a ratio of 1:1. After baseline measurements, each patient was administered 300 mL of glucose agent containing 75 g of glucose. Blood samples were collected using an intravenous catheter (SAFELET CATH; Nipro, Osaka, Japan), t‐type extension tubes (T‐type Extension Tube; Perfect Medical Industry, Ho Chi Minh City, Vietnam), and sterile syringes (5‐mL slip‐tip disposable syringes and BD PrecisionGlide Needle‐23G; BD, Temse, Belgium).

### 2.11. Measurement of Liver and Kidney Functions

The patients′ blood samples were analyzed by Lezen Reference Laboratory. Collection tubes and a centrifuge (CN‐830; Hsiang Tai Machinery Industry, New Taipei, Taiwan, China) were kindly provided by Lezen Reference Laboratory. To measure the indicators of liver and kidney functions, blood samples were collected in Weeks 0 and 8. To measure the indicators of T2DM, blood samples were collected in Weeks 0, 4, and 8. For the measurement of AST, ALT, ALP, CRE, and insulin, whole‐blood samples were collected by using vacutainer tubes spray‐coated with silica and a polymer gel; the collected samples were set aside for 30 min and then centrifuged at 3500 rpm for 10 min to separate serum from blood cells. AST, ALT, ALP, and CRE were measured through colorimetric assays, whereas insulin was measured through a chemiluminescent microparticle immunoassay. For the measurement of HbA1c, whole‐blood samples were collected using vacutainer tubes spray‐coated with tripotassium ethylenediaminetetraacetic acid. HbA1c was measured through affinity chromatography. For the measurement of blood glucose levels, whole‐blood samples were collected using vacutainer tubes spray‐coated with sodium fluoride. Blood glucose levels were measured through colorimetric assays.

### 2.12. Statistical Analysis

Data are presented in terms of the mean ± standard deviation (SD) values. The significance of between‐group was evaluated through analysis of variance (ANOVA). Nonparametric variables were compared using the Wilcoxon signed‐rank test. A *p* value of < 0.05 was considered to be statistically significant.

## 3. Results

### 3.1. Survival and Clinical Signs of Mice

As per our study design, we used 120 mg/day/kg body weight of TF601 polyherbal drug to evaluate the toxic effect on the mice model. None of the mice orally exposed to 120 mg/day/kg died. No changes in posture, handling response, clonic or tonic movement, or behavior were noted in any group. An ophthalmological examination performed at the end of the study showed no abnormalities in TF601 polyherbal drug–treated male and female group mice during the study.

### 3.2. Effect of TF601 on Body Weight and Food Intake of Mice

During this study, the body weight of mice was increased following the usual pattern of this species. After TF601 polyherbal drug treatment, the body weight of male and female mice increased over the 90‐day period, but the increase was lower in male mice than in female mice (Figure 1a). Compared with the control mice, the treated female mice exhibited an increase in body weight (Figure 1b).

Figure 1Effect of TF601 on body weight and metabolic parameters in experimental mice. (a, b) Time‐dependent changes in body weight and (c, d) metabolic parameters in control and TF601‐treated mice over the experimental period. Data are expressed as mean ± SD (*n* = 5 per group)  ^∗^ *p* < 0.05,  ^∗∗^ *p* < 0.01,  ^∗∗∗^ *p* < 0.001, and ns (nonsignificant).

**Figure:**
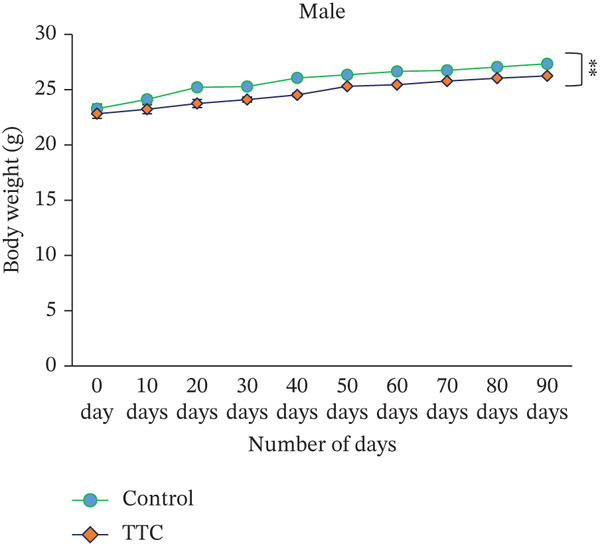
(a)

**Figure:**
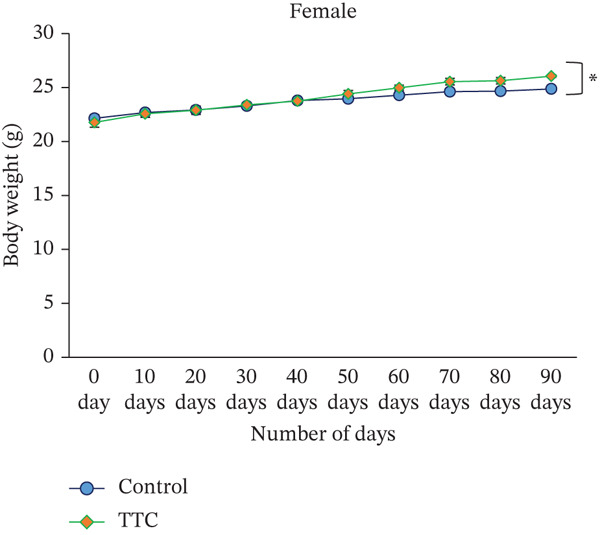
(b)

**Figure:**
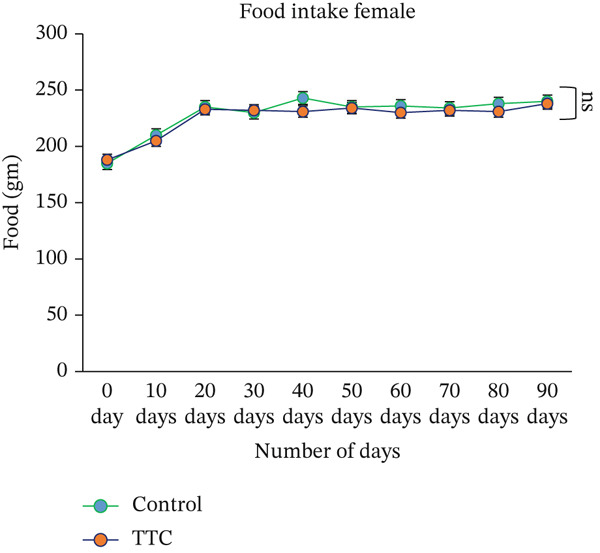
(c)

**Figure:**
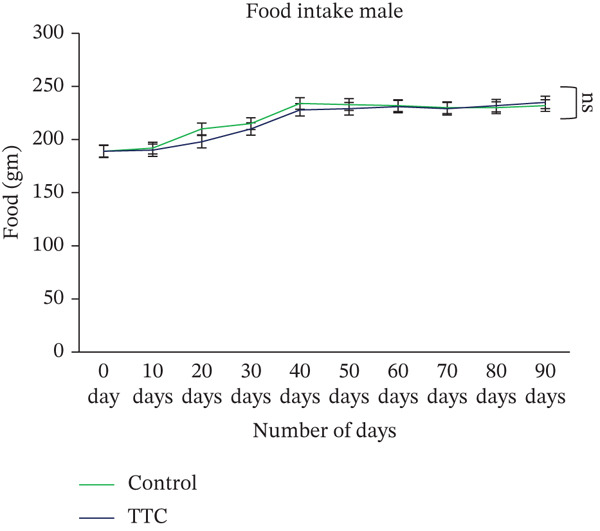
(d)

Throughout the study period, no significant difference was noted between the control and treatment groups (either male or female) in weekly food intake (Figure 1c,d). Feed consumption did not reveal any changes in animals.

### 3.3. Hematology and White Blood Cell Counts After Oral Administration of TF601 in Mice

Hematological parameters were evaluated to assess the potential effects of repeated oral administration of TF601 on erythropoiesis and oxygen‐carrying capacity (Table 1). The analyzed parameters included HCT, RBC count, hemoglobin (Hb), and MCV, and their quantitative data are presented as bar graphs (Figure 2) As shown in Figure 2, no statistically significant differences were observed in RBC count between the control and TF601‐treated groups. Similarly, HGB concentration, HCT values, and MCV, remained comparable between groups, indicating that TF601 administration did not affect overall erythrocyte mass or oxygen‐transport capacity. Overall, these findings indicate that TF601 does not exert adverse effects on RBC production, morphology, or Hb synthesis under the conditions of this study.

**Table 1:** Hematology parameters of male and female mice fed with 120 mg/kg body weight/day TF601 for 90 days. Values are mean ± SD for *n* = 5 mice/sex/group. The difference between treated and control groups of male and female mice was evaluated by ANOVA test.

	Male				Female			
	CTRL		TTC (120 mg/mL)		CTRL		TTC (120 mg/mL)	
Parameters	Mean	± SD	Mean	± SD	Mean	± SD	Mean	± SD
RBC count (M/*μ*L)	9.03	0.8469	9.614	0.2656	9.52	0.6793	9.224	0.8406
HGB (g/dL)	12.88	1.0378	13.44	0.3286	13.44	0.9423	13.22	1.1345
HCT value (%)	44.3	3.1953	46	1.5166	44.8	2.947	44.58	3.5982
MCV value (fL)	49.14	1.274	47.86	0.7925	47.08	0.712	48.4	1.7986
MCH value (pg)	14.26	0.3435	13.98	0.0837	14.12	0.0837	14.34	0.5413
MCHC value (g/dL)	29.06	0.2702	29.2	0.4183	29.98	0.3633	29.64	0.1517
RDW‐CV (%)	24.06	1.5534	23.08	1.0232	22.7	0.2915	22.94	1.3069
RET count (K/*μ*L)	778.46	505.2537	444.68	54.5548	437.22	90.9349	553.76	135.7569
RET value (%)	9.106	6.6433	4.62	0.4913	4.654	1.2483	6.124	2.0118
PLT count (10^9^/L)	1445.8	79.8417	1161	172.5036	1122.6	396.0212	1092.4	164.3375
MPV value (fL)	8.76	0.4037	8.5	0.2121	8.94	0.2881	8.7	0.495
PCT value (%)	1.266	0.0876	0.988	0.1492	0.998	0.346	0.942	0.1469
PDW value (fL)	6.56	0.2702	6.76	0.305	6.68	0.3899	6.8	0.2345

Figure 2Effect of TF601 on hematological parameters in experimental animals. Showing selected hematological parameters, including (a) hematocrit (HCT), (b) red blood cell count (RBC), (c) hemoglobin (HGB), and (d) mean corpuscular volume (MCV), in control and TF601‐treated groups. Data are presented as mean ± SD (*n* = 5 animals per group)  ^∗^ *p* < 0.05,  ^∗∗^ *p* < 0.01,  ^∗∗∗^ *p* < 0.001, and ns (nonsignificant).

**Figure:**
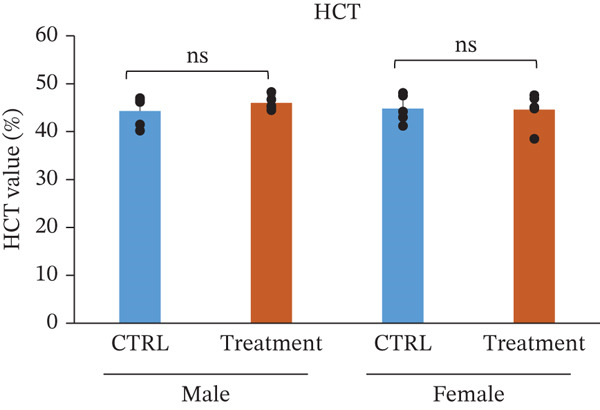
(a)

**Figure:**
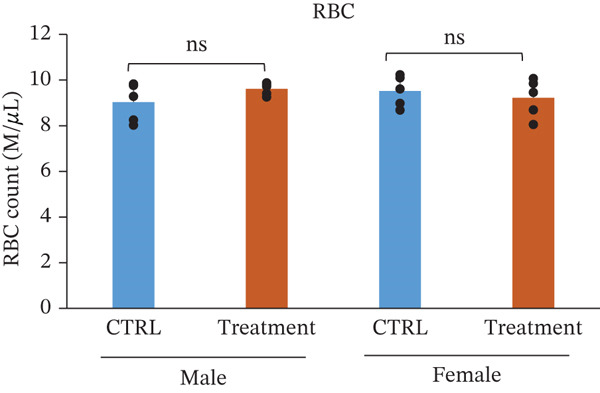
(b)

**Figure:**
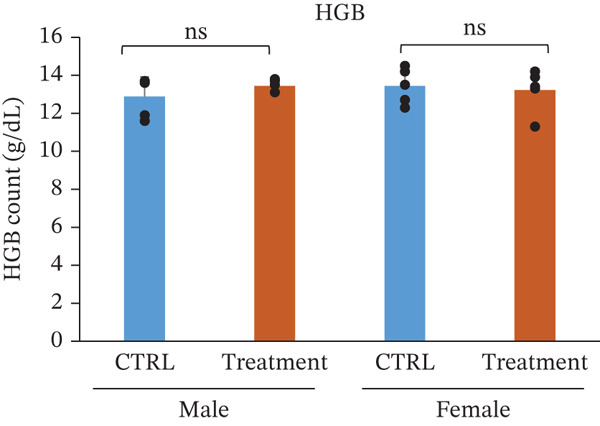
(c)

**Figure:**
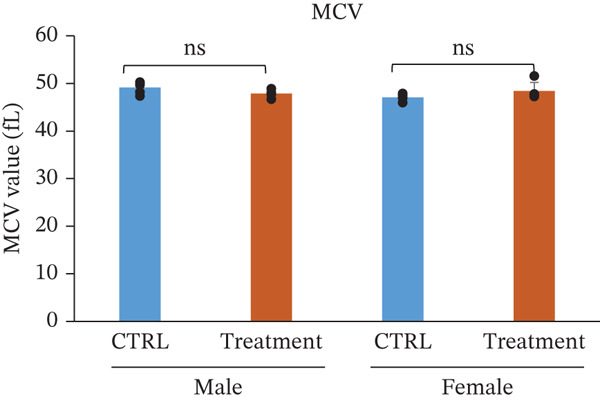
(d)

White blood cell parameters were analyzed to evaluate the potential immunological and inflammatory effects of TF601 (Table 2). Total white blood cell counts and differential leukocyte profiles, including NEU, LYM, and MONO, are presented in Figure 3. Total white blood cell counts did not differ significantly between the control and TF601‐treated groups in female mice. In contrast, male mice exhibited a modest but statistically significant alteration in total white blood cell counts following TF601 treatment. Differential leukocyte analysis revealed a significant reduction in neutrophil counts in TF601‐treated male mice compared with control males, whereas no significant changes in neutrophil levels were observed in female mice. LYM and MONO counts remained comparable between the control and TF601‐treated groups in both sexes, with all values remaining within normal physiological ranges.

**Table 2:** Differential white blood cell count data of male and female mice fed with 120 mg/kg body weight/day TF601 for 90 days. Values are mean ± SD for five mice/sex/group. The difference between treated and control groups of male and female mice was evaluated by ANOVA test.

	Male				Female			
	CTRL		TTC (120 mg/mL)		CTRL		TTC (120 mg/mL)	
Parameters	Mean	± SD	Mean	± SD	Mean	± SD	Mean	± SD
WBC count (k/*μ*L)	6.682	1.4041	4.988	0.8486	5.146	2.1854	5.688	0.866
NEU count (K/*μ*L)	1.59	0.4259	0.932	0.2698	0.944	0.5913	0.556	0.1845
LYM count (k/*μ*L)	4.904	1.1462	3.89	1.0783	4.008	1.6875	4.798	0.9727
MONO count (k/*μ*L)	0.094	0.0321	0.078	0.0492	0.11	0.0616	0.288	0.4823
EOS count (k/*μ*L)	0.092	0.0804	0.078	0.0249	0.136	0.1313	0.072	0.0228
BASO count (k/*μ*L)	0.002	4.00E‐03	0.01	0.01	0.002	4.47E‐03	0.01	0
NEU %	24.12	6.6972	19.8	8.681	17.5	6.181	9.64	2.4896
LYM %	73.22	6.2683	76.84	8.9634	78.14	6.0107	83.92	10.5222
MONO %	1.4	0.3808	1.62	1.0281	2.24	1.6727	5	8.3869
EOS %	1.22	0.8871	1.52	0.3033	2.04	1.8555	1.26	0.2966
BASO %	0.04	0.0894	0.22	0.228	0.08	0.1789	0.18	0.0447

Figure 3Effect of TF601 on white blood cells parameters in TF601‐treated mice. Graphs showing selected parameters, (a) white blood cell count (WBC), (b) neutrophils (NEU), (c) monocytes (MONO), and (d) lymphocytes (LYM), in control and TF601‐treated groups. Data are presented as mean ± SD (*n* = 5 animals per group)  ^∗^ *p* < 0.05,  ^∗∗^ *p* < 0.01,  ^∗∗∗^ *p* < 0.001, and ns (nonsignificant).

**Figure:**
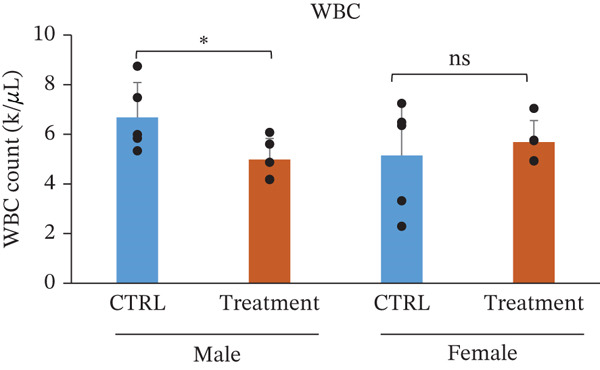
(a)

**Figure:**
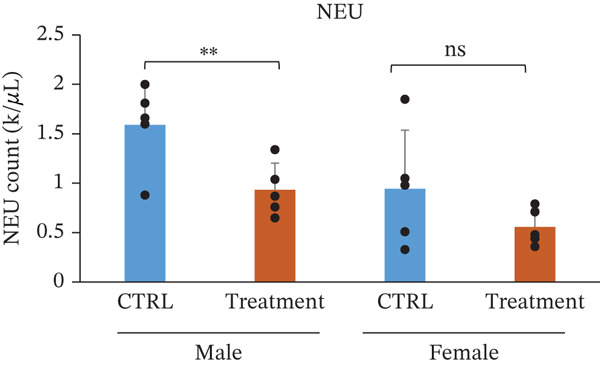
(b)

**Figure:**
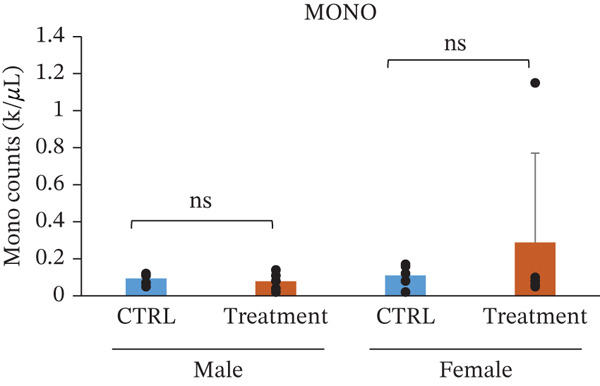
(c)

**Figure:**
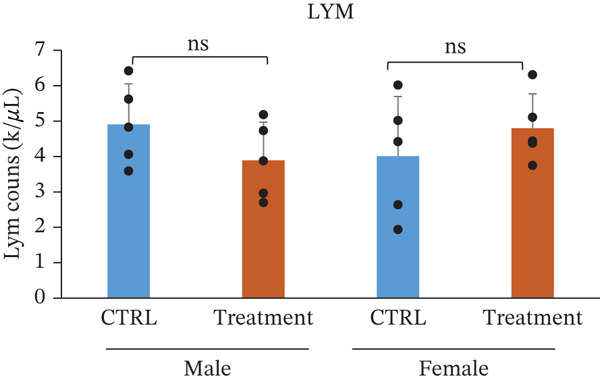
(d)

Overall, these findings indicate that TF601 administration resulted in a mild, sex‐dependent alteration in neutrophil levels without evidence of overt inflammatory responses or immunosuppressive effects. The observed changes were limited in magnitude and did not disrupt overall leukocyte homeostasis in mice.

### 3.4. Blood Biochemistry of Mice

Serum biochemical parameters were evaluated to assess the potential effects of TF601 on hepatic and renal function (Table 3). The analyzed parameters included glutamate oxaloacetate transaminase (GOT/AST), ALP, BUN, CRE, and their quantitative data are presented in Figure 4. Serum GOT (AST) levels showed no significant differences between the control and TF601‐treated groups in male or female mice. The absence of elevated transaminase activity supports the lack of TF601‐induced hepatocellular injury. Similarly, ALP levels did not differ significantly between the control and TF601‐treated groups in either male or female mice, indicating the absence of treatment‐related alterations in hepatobiliary function. Renal function markers, including serum CRE and BUN, remained comparable between the control and TF601‐treated groups in both sexes. No statistically significant changes were observed, and all measured values were within normal physiological ranges, suggesting that TF601 administration did not adversely affect renal function. Collectively, these biochemical findings indicate that repeated oral administration of TF601 for 90 days does not induce clinically relevant hepatic or renal dysfunction in mice.

**Table 3:** Blood chemistry of male and female mice fed with 120 mg/kg body weight/day TF601 for 90 days. Values are mean ± SD for five mice/sex/group. The difference between treated and control groups of male and female mice was evaluated by ANOVA test.

	MALE				FEMALE			
	CTRL		TTC (120 mg/mL)		CTRL		TTC (120 mg/mL)	
Parameters	Mean	± SD	Mean	± SD	Mean	± SD	Mean	± SD
GOT (U/l)	124.4	52.5338	62.4	44.3091	279.5	273.7742	185.5	201.6226
GTP (U/l)	76.6	43.322	29.8	6.7231	223	306.0654	101.25	121.5027
ALP (U/l)	166.2	33.6853	157	24.2074	208	82.668	220.75	21.5619
Glu	439.6	141.5037	470	77.4016	276.25	93.3073	175.5	29.2176
BUN	23.98	4.3854	26.56	0.9788	24.8	8.1744	20.625	3.7836
CRE	0.234	0.0699	0.276	0.0559	0.165	0.0705	0.1075	0.0359
UA	1.9	0.5788	1.48	0.2775	1.525	0.6994	1.75	0.4509
TCHO	68.2	12.8725	57.096	32.0526	57.5	2.3805	57.75	11.8708
TG	110.6	53.271	114.8	25.5284	44.25	10.0125	19	6.4807
TBIL	0.7	0.0707	0.68	0.1095	0.8	0.0816	0.675	0.0957
TP	4.32	0.3271	4.12	0.228	4.275	0.2986	4.325	0.3775
ALB	2.1	0.255	1.88	0.1304	2.1	0.2449	2.175	0.263
IP	9.84	1.8188	9.32	0.7294	9.55	2.7683	9.1	2.0347
Ca	7.58	0.3033	7.52	0.4658	7.5	0.3742	7.35	0.526
Na	148.8	5.1186	149	5	148.25	3.4034	152.25	3.304
K	4.66	0.3435	5.22	0.531	5.45	0.6455	4.5	0.6976
Cl	108.6	6.5803	110.8	5.6303	109.75	4.1932	111.5	2.6458

Figure 4Effect of TF601 on serum biochemical parameters. Graphs representing key serum biochemical markers associated with hepatic and renal function, including (a) glutamate oxaloacetate transaminase (GOT), (b) alkaline phosphatase (ALP), (c) blood urea nitrogen (BUN), and (d) creatinine (CRE), in control and TF601‐treated groups. Data are expressed as mean ± SD (*n* = 5 animals per group)  ^∗^ *p* < 0.05,  ^∗∗^ *p* < 0.01,  ^∗∗∗^ *p* < 0.001, and ns (nonsignificant).

**Figure:**
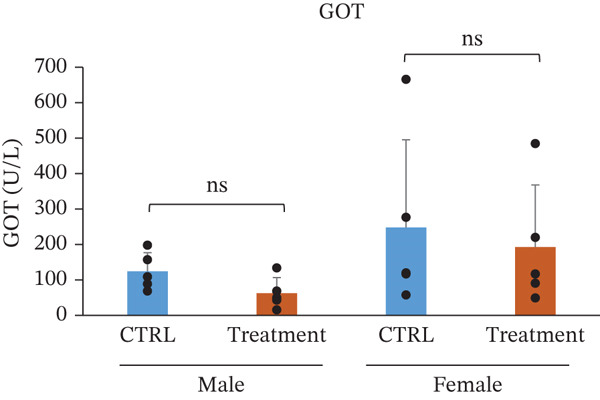
(a)

**Figure:**
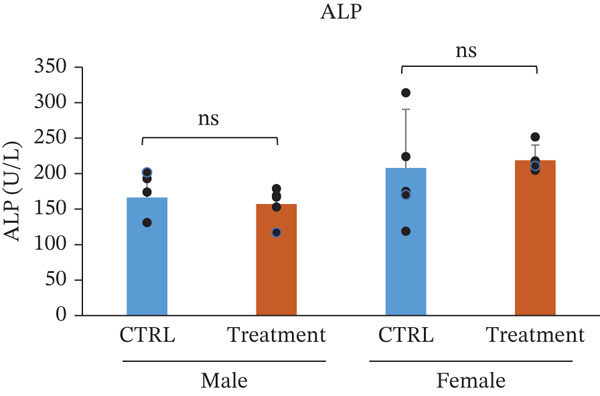
(b)

**Figure:**
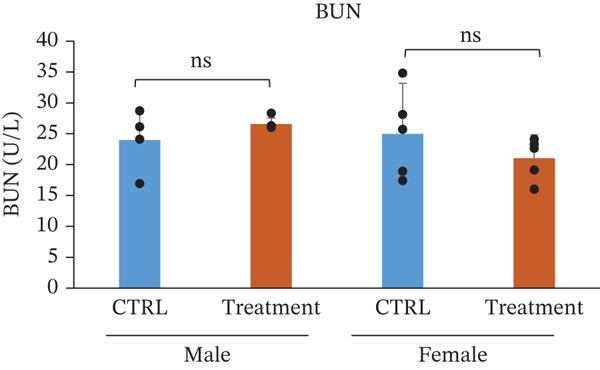
(c)

**Figure:**
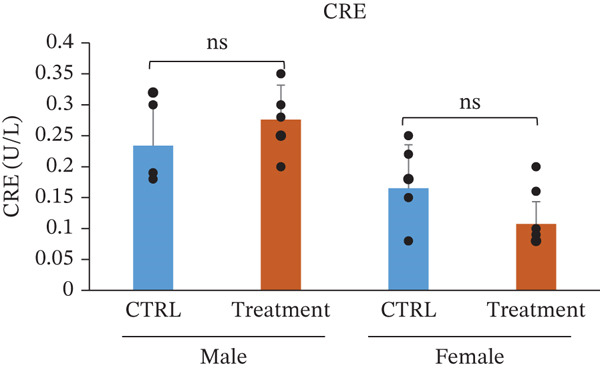
(d)

### 3.5. Histopathological Examination of Mice After 90 Days of TF601 Administration

**Figure 5:**
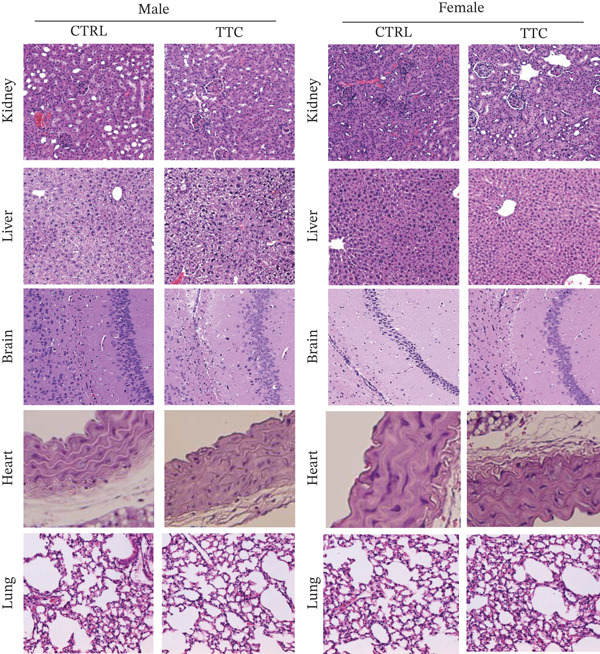
Histopathological evaluation of major organs following TF601 treatment. Representative hematoxylin and eosin (H&E)–stained sections of liver, kidney, brain, heart, lung from control and TF601‐treated animals.

Histopathological examination was performed in accordance with OECD Test Guideline 408, which emphasizes qualitative microscopic examination to identify treatment‐related lesions, with severity grading applied when pathological changes are observed. Therefore, we examine the major organs, including the kidney, liver, brain, heart, and lung, to evaluate potential tissue‐level toxicity following repeated oral administration of TF601 for 90 days. Representative H&E‐stained sections from TF601‐treated and control animals demonstrated largely preserved tissue architecture across all examined organs (Figure 5). Kidney sections exhibited intact glomeruli and renal tubules without evidence of tubular degeneration, glomerular damage, or interstitial inflammation. Liver sections showed preserved hepatic architecture with well‐organized hepatocyte cords and intact sinusoids. Occasional mild variations in hepatocyte nuclear morphology were observed; however, these findings were minimal, nonprogressive, and also present in control sections, and were therefore considered incidental rather than treatment related. Brain sections revealed intact neuronal morphology and preserved cortical/hippocampal architecture without evidence of neuronal degeneration, gliosis, or vacuolation. Moreover, cardiac tissues displayed normal myocardial organization with well‐aligned fibers and preserved striations, and no signs of myofiber degeneration or inflammatory infiltration were observed. In addition, lung sections also demonstrated normal pulmonary architecture with intact alveolar spaces and septa, without evidence of edema, hemorrhage, or inflammatory cell infiltration. Overall, no treatment‐related histopathological alterations were identified in any of the examined organs following 90‐day oral exposure to TF601.

### 3.6. Type 2 Diabetic Patient Enrollment for Clinical Trial of TF601

Between September 2021 and August 2022, we screened 32 patients. From them, 18 were excluded as they did not meet our inclusion criteria. Finally, 14 patients were included (Figure 6).

Figure 6Flowchart depicting patient enrollment. (a) Participant selection. (b) Of a total of 14 patients with T2DM, 8 were men and 6 were women.

**Figure:**
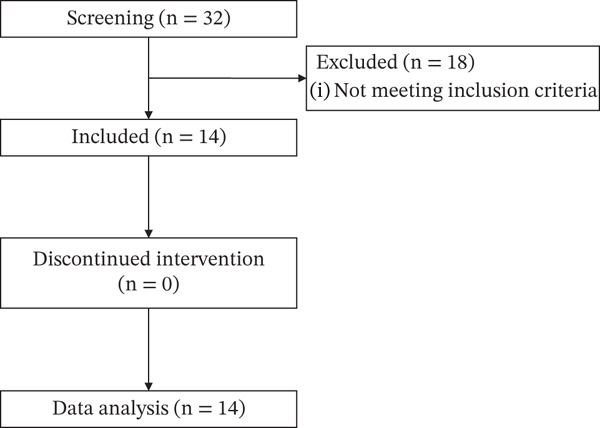
(a)

**Figure:**
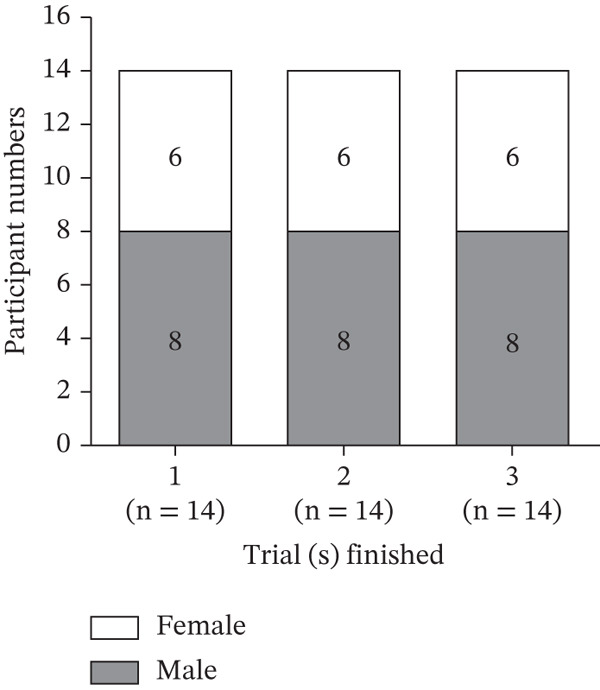
(b)

### 3.7. Baseline Characteristics

In Week 0, the mean age of the participants was 57.71 ± 8.32 years (men: 54.88 ± 7.45 years; women: 61.50 ± 8.50 years). Six patients (four men and two women) had T2DM for < 5 years, four (two men and two women) had T2DM for 6–10 years, and four (two men and two women) had T2DM for > 10 years. All participants received oral medications for T2DM; one medicine, one man; two medicines, three men and five women; and three medicines, four men and one woman. The mean body weight of the participants was 65.66 ± 13.09 kg (men: 72.66 ± 11.64 kg; women: 56.32 ± 8.52 kg). The mean HbA1c percentage of the participants was 7.66*%* ± 0.72*%* (men: 7.98*%* ± 0.83*%*; women: 7.25*%* ± 0.16*%*). As per the OGTT results, the participants′ mean FBG level was 129.64 ± 27.79 mg/dL, mean fasting insulin level was 10.48 ± 8.05 *μ*U/mL, and mean 2hBG level was 273.71 ± 47.07 mg/dL (Table 4).

**Table 4:** Baseline characteristics of the participants.

	Men	Women	Total
**Demographic characteristics**			
Age (years)	54.88 ± 7.45	61.50 ± 8.50	57.71 ± 8.32
Patient count	8	6	14

Diabetes duration (years)			
0–5	4	2	6
6–10	2	2	4
> 11	2	2	4

Number of oral hypoglycemic agents used			
0	0	0	0
1	1	0	1
2	3	5	8
3	4	1	5

Anthropometric characteristics			
Height (cm)	166.69 ± 3.60	156.33 ± 4.84	162.25 ± 6.65
Weights (kg)	70.88 ± 12.83	56.32 ± 8.52	63.60 ± 12.87
BMI (kg/m^2^)	25.19 ± 3.63	23.03 ± 3.23	24.11 ± 3.46

Laboratory data			
HbA1c (%)	7.98 ± 0.83	7.25 ± 0.16	7.66 ± 0.72
FBG (mg/dL)	120.25 ± 18.38	142.17 ± 34.72	129.64 ± 27.79
Fasting insulin (*μ*U/mL)	11.60 ± 10.00	8.98 ± 4.85	10.48 ± 8.05
HOMA‐IR	3.68 ± 3.38	3.48 ± 2.55	3.46 ± 2.83
2hBG (mg/dL)	262.13 ± 54.08	289.17 ± 34.13	273.71 ± 47.07
AST (U/L)	20.13 ± 6.17	17.83 ± 7.70	19.14 ± 6.69
ALT (U/L)	25.25 ± 15.13	16.17 ± 7.41	21.36 ± 12.89
ALP (U/L)	47.25 ± 11.68	55.67 ± 13.94	50.86 ± 12.92
Creatinine (mg/dL)	0.98 ± 0.20	0.60 ± 0.09	0.82 ± 0.25
eGFR (mL/min/1.73 m^2^)	87.81 ± 19.95	110.44 ± 17.27	97.51 ± 21.54

*Note:* Data are presented in terms of the number of patients (*n*) or mean ± SD values.

Abbreviations: 2hBG, 2‐h blood glucose; ALP, alkaline phosphatase; ALT, alanine aminotransferase; AST, aspartate aminotransferase; BMI, body mass index; eGFR, estimated glomerular filtration rate; FBG, fasting blood glucose; HbA1c, hemoglobin A1c; HOMA‐IR, homeostatic model assessment of insulin resistance.

### 3.8. FBG Levels, Liver, and Kidney Functions in T2DM Patients After TF601 Treatment

One HbA1c data point pertaining to Week 4 was found to be an outlier; this was removed through the ROUT method. The mean HbA1c levels were found to be significantly reduced in Week 4 (7.35*%* ± 0.96*%* and 7.32*%* ± 0.70*%*). No significant changes from baseline were observed in FBG levels (117.50 ± 17.53 mg/dL and 123.64 ± 19.15 mg/dL, respectively) or fasting insulin levels (9.72 ± 4.13 *μ*U/mL and 12.35 ± 10.08 *μ*U/mL, respectively). Therefore, the homeostatic model assessment of insulin resistance index remained unchanged (2.82 ± 1.32 and 3.71 ± 2.95, respectively). Significant reductions in 2hBG levels were noted in Week 4 (248.00 ± 49.18 mg/dL) but not in Week 8 (262.29 ± 68.48 mg/dL). In Week 8, no significant changes were observed in the indicators of liver function (AST, ALT, and ALP) or kidney indicators (CRE and eGFR; Table 5). Notably, two men did not return the data collection forms at the end of the trial; therefore, we analyzed the data (Weeks 1–8) of six men and six women. During the 8‐week trial, no significant changes were observed in patients′ body weight or BMI (Figure 7a,b). Although no significant changes were noted in FBG levels compared with baseline levels, bedtime blood glucose (BBG) levels exhibited significant reductions in Weeks 6 and 8 (Figure 8a,b).

**Table 5:** Patients′ biochemical test after 4 and 8 weeks of TF601 treatment.

Parameters	Week 4			Week 8		
	Patient count	Value	*p* value^a^	Patient count	Value	*p* value^b^
HbA1c (%)	13	7.35 ± 0.96	0.0012 ^∗∗^	14	7.32 ± 0.70	0.0005 ^∗∗∗^
FBG level (mg/dL)	14	117.50 ± 17.53	0.2225	14	123.64 ± 19.15	0.9214
Fasting insulin (*μ*U/mL)	14	9.72 ± 4.13	0.9032	14	12.35 ± 10.08	0.5016
HOMA‐IR	14	2.83 ± 1.32	0.7609	14	3.71 ± 2.95	0.6257
2hBG (mg/dL)	14	248.00 ± 49.18	0.0007 ^∗∗∗^	14	262.29 ± 68.48	0.6698
AST (U/L)	14	—	—	14	18.86 ± 5.49	0.8301
ALT (U/L)	14	—	—	14	18.57 ± 8.13	0.1235
ALP (U/L)	14	—	—	14	48.36 ± 13.90	0.1777
Creatinine (mg/dL)	14	—	—	14	0.78 ± 0.26	0.1477
eGFR (mL/min/1.73 m^2^)	14	—	—	14	105.57 ± 30.58	0.1189

*Note:* Data are presented in terms of the number of patients (*n*) or mean ± SD values and were compared using the Wilcoxon signed‐rank test.

Abbreviations: 2hBG, 2‐h blood glucose; ALP, alkaline phosphatase; ALT, alanine aminotransferase; AST, aspartate aminotransferase; BMI, body mass index; eGFR, estimated glomerular filtration rate; FBG, fasting blood glucose; HbA1c, hemoglobin A1c; HOMA‐IR, homeostatic model assessment of insulin resistance.

^a^ *p* value of Week 4 versus Week 0.

^b^ *p* value of Week 8 versus Week 0.

^∗∗^ *p* < 0.01 versus Week 0;  ^∗∗∗^ *p* < 0.001 versus Week 0.

Figure 7Weekly changes in patients′ body weight and BMI over an 8‐week period. (a) Body weight data. In Week 3, a significant change was observed in body weight. (b) BMI data. Significant changes were observed in BMI in Week 3.  ^∗^ *p* < 0.05,  ^∗∗^ *p* < 0.01,  ^∗∗∗^ *p* < 0.001, and ns (nonsignificant).

**Figure:**
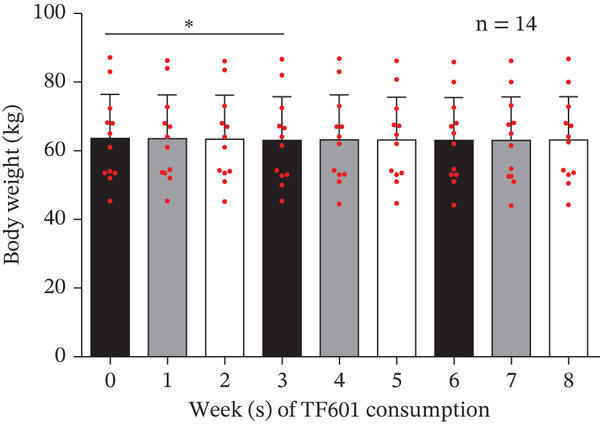
(a)

**Figure:**
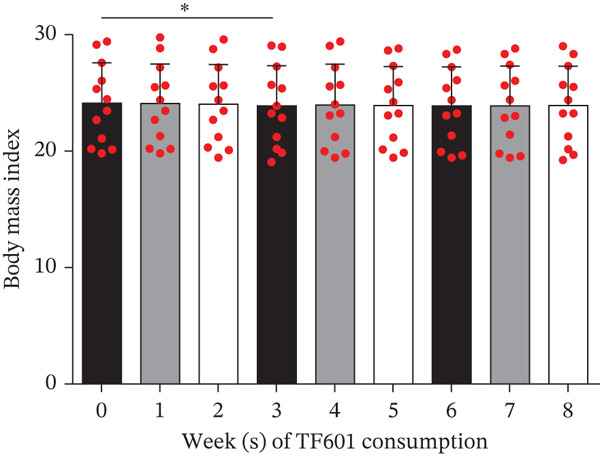
(b)

Figure 8Daily changes in patients′ fasting and bedtime blood glucose levels over an 8‐week period. (a) Fasting blood glucose data. No significant change was observed during the trial. (b) Bedtime blood glucose data. Significant changes were observed in Weeks 6 and 8 of the trial.  ^∗^ *p* < 0.05,  ^∗∗^ *p* < 0.01,  ^∗∗∗^ *p* < 0.001, and ns (nonsignificant).

**Figure:**
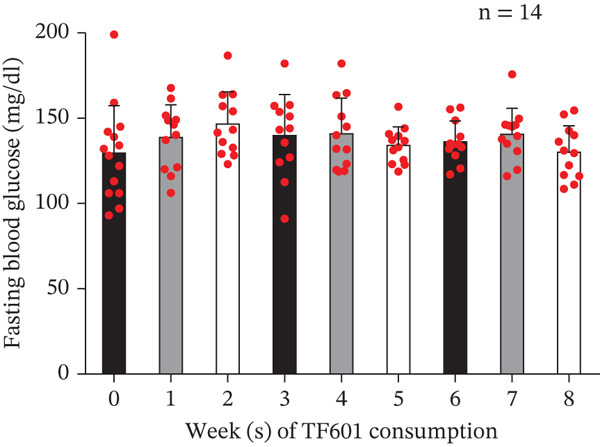
(a)

**Figure:**
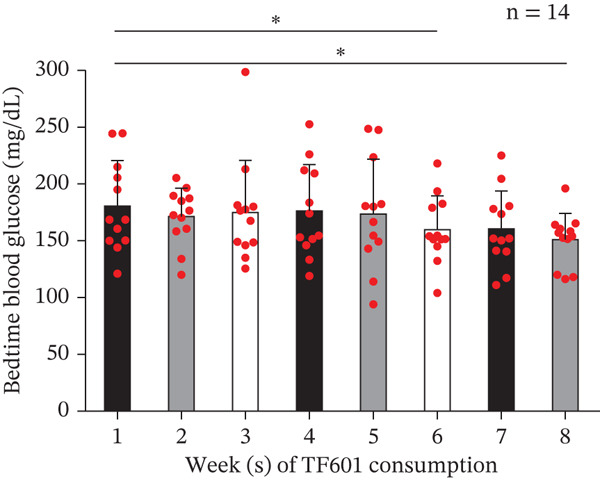
(b)

## 4. Discussion

Polyherbal products have long been used worldwide for the treatment of various human diseases. Generally, it is assumed that polyherbal drugs and their products are nontoxic, without adverse effects, and safe. However, several studies have reported that all polyherbal extracts also exert some adverse effects, resulting from the inherent toxic effects of active components, overdosing, long‐term use, and are completely not safe [7, 20]. Therefore, the toxicological properties of polyherbal products must be evaluated to ensure patient safety.

Notably, repeated 90‐day–oral toxicity studies in mice can reveal the adverse effects likely to arise from repeated dose exposure over a prolonged period covering growth into adulthood of the test animals [21]. This is the first toxicity study of a polyherbal thirst‐free drink TF601 and contains the cocktail of herbal plant aqueous extracts. Components in TF601 polyherbal drink have been reported to have antioxidative, antidiabetic, and antiarteriosclerotic properties [2, 12]. *P. guajava* acts as an antioxidant and antidiabetic. Aqueous extracts of *P. guajava* improve hepatic insulin sensitivity by promoting glycogen synthesis and inhibiting gluconeogenesis [22]. These extracts can also upregulate GLUT2 expression and promote glucose uptake, thereby improving glucose metabolism in patients with T2DM [17]. Several studies have reported the anti‐inflammatory and antioxidative properties of *P. guajava* [23]. A recent in silico study demonstrated that *P. guajava* can protect against gastric ulcers [24]. It is widely demonstrated that *M. charantia* extracts inhibit glycemic conditions in human and animal models of T2DM [25]. Most studies on *M. charantia* have been performed on cellular and animal models. Both in vitro and in vivo studies have reported toxic and adverse effects *M. charantia* extracts under certain conditions [26]. An animal study investigating the toxicity of *M. charantia* reported that a dose of < 2000 mg/kg of exerted no toxic effect [27]. *Lycii Fructus* mitigates cytotoxicity by upregulating the antioxidant gene expressions [28]. It also improves renal function in obese mice and exerts antiaging, anticancer, and hypolipidemic effects [29]. Thus, each component of TF601 confers medicinal benefits and is safe. However, their combined effects remain to be investigated.

Body weight loss, mortality, altered food intake, and severe clinical signs are essential indicators of a drug component′s adverse effects in animal models [30]. In our study, we did not find any TF601‐related deaths or severe clinical signs. The normal food intake observed in the treatment groups indicated that TF601 intake does not alter metabolic profiles. These findings are consistent with those reported by a study investigating a polyherbal formulation containing extracts from *Allium sativum*, *Terminalia* sp., *Curcuma* sp., and *Amomum* sp., in which the formulation did not affect the body weight or food intake of rats [31]. We found that a gradual increase in mouse body weight increment over the study period was observed, likely because of the nutrients component of TF601. Overall, TF601 did not alter body weight or food intake in male or female mice.

In toxicity studies, significant changes in blood parameters often result from the adverse effects of test compounds [8, 32]. In our study, the hematological parameters of TF601‐treated mice were similar to those of the control mice. However, only male mice exhibited slight reductions in reticulocyte and MCH counts, indicating a very mild effect of TF601 on the liver. *M. charantia* fruits extract can prevent gastric, stress‐induced ulcers in animal models [33]. Several studies have also reported the antiulcer activity of *P. guajava* [24, 34].

In our study, blood serum analyses revealed that no significant differences existed between TF601‐treated and control mice. Furthermore, no significant pathological lesions were found in histological tissues of the kidney, liver, and brain tissues of TF601‐treated mice. Only mild hepatic lesions were observed in all groups, including the control groups. However, these lesions did not affect the hepatocytes, as evident from the fact that the levels of liver‐specific enzymes AST and ALT decreased slightly in both treatment groups compared with the levels in the control groups.

Food consumption, body weight, blood biochemistry, and hematological parameters indicated sex‐dependent changes. For some parameters, the values were lower in female mice than in male mice, perhaps because of the sex‐based differences in drug metabolism due to the presence of different hormones, fatty acids, and enzymes [35]. For instance, the percentage of enzyme cytochrome P450, which participates in drug metabolism and helps in the excretion of drugs, is higher in male rodents than in female rodents [36]. Thus, male and female mice had different responses to the drug treatment, which supports the current findings.

Various phytochemicals have been reported to be effective against T2DM [37, 38]. Triterpenoids present in *M. charantia* may help achieve glycemic control by enhancing the activity of adenosine monophosphate–activated protein kinase [39, 40]. *P. guajava* contains quercetins and exert anti‐inflammatory effects through the downregulation of interleukin‐1*β*, interleukin‐18, and NLRP3 [41]. To date, among the ingredients of TF601, only *M. charantia* has entered clinical trials involving patients with T2DM. Compared with other T2DM studies on *M. charantia* [42] to the best of our knowledge, this study is the first to investigate the antidiabetic effects of a polyherbal drink in human. The mean HbA1c levels of our participants exhibited significant reductions in Weeks 4 and 8. According to self‐measurement data, compared with baseline levels, FBG levels were significantly reduced in Week 8 (*p* = 0.0425) and BBG levels were significantly reduced in Weeks 6 and 8 (*p* = 0.0200 and 0.0283, respectively) but not in Week 7 (*p* = 0.0640). According to hospital‐based measurements, compared with baseline levels, FBG levels remains unchanged in Weeks 4 and 8 and 2hBG levels exhibited significant reductions in Week 4 (*p* = 0.0007) but not in Week 8. These findings suggest that TF601 exerts hypoglycemic effects. In most of our participants, the baseline percentage of HbA1c was 7.0%–8.0%; this percentage eventually decreased to 7.0%–7.5% in Week 8, with the lowest data point still remaining in the normal range. After the 8‐week TF601 trial, no significant changes were observed in the patients′ weekly BMI data; AST, ALT, ALP, or CRE levels; or eGFR. However, glycemic indices were significantly affected, but the values were still within the corresponding normal ranges. These findings confirm the safety of TF601. In our study, BBG levels exhibited significant reductions in Week 6, which may explain the ameliorated FBG levels in Week 8. Our study has several limitations. First, we did not include a control group in our clinical trial. Second, the human component was designed as an exploratory, uncontrolled pilot study with a small sample size (*n* = 14), which limits statistical power and generalizability. In addition, only a single‐dose level was evaluated in the animal toxicity study, and mechanistic biomarkers related to oxidative stress, inflammation, or insulin sensitivity were not assessed, and also the participants received no dietary instructions to follow during the trial period; they had different lifestyles, which might have introduced bias. Different dietary patterns can differentially influence insulin resistance or HbA1c percentage in patients with T2DM [43–45]. In this present study, histopathological evaluation was qualitative in nature, and quantitative lesion scoring or blinded pathological assessment was not performed. Moreover, histological examination of spleen was not performed; however, no hematological or leukocyte abnormalities indicative of immune toxicity were observed, supporting the overall toxicological profile of TF601 under the study conditions. Therefore, the findings should be interpreted as preliminary and hypothesis‐generating, warranting confirmation through dose–response studies and randomized controlled clinical trials.

## 5. Conclusion

TF601 did not exert any major toxic effects in our animal model, as evident from the food intake patterns, body weight, blood biochemistry, hematological parameters, and histopathological parameters. Only mild pathological changes were detected in the liver. Long‐term toxicity studies are warranted to investigate the inconsistency between the histopathological and biochemical findings. Our findings indicate that TF601 is generally safe, particularly at low concentrations (< 120 mg/mL). Further preclinical animal studies (chronic, genotoxicity, and developmental toxicity) are required to gather sufficient evidence for its use in humans. Monitoring multiple indicators revealed the antidiabetic effects of TF601. This drink may serve as an adjunct treatment option for patients with T2DM. Future studies are warranted to investigate the effects of TF601 on HbA1c percentage.

## Author Contributions

**Tai-Chi Hung and Yue-Hua Deng:** conceptualization, methodology, data curation, and investigation. **Yu-Hsin Hung:** investigation, methodology, and writing—original draft. **Win-Ping Deng:** writing—review and editing. **Fang-Pey Chen:** formal analysis and data curation. **Hong-Jian Wei:** investigation. **Abhinay Kumar Singh:** investigation, methodology, and writing—original draft. **Chi-Sheng Chiou:** supervision, resource, writing—review and editing, and funding acquisition. **Tai-Chi Hung** and **Yue-Hua Deng** have contributed equally to this work.

## Funding

This study was supported by the Stem Cell Research Center, Taipei Medical University (TMU112‐AE1‐B06), National Science and Technology Council Taiwan (NSTC 112‐2221‐E‐038‐019), and Wan Fang Hospital, Taipei Medical University (111‐wf‐f‐6).

## Ethics Statement

All animal experimental protocols have been carried out in accordance with relevant guidelines and all methods are reported in accordance with ARRIVE guidelines (https://arriveguidelines.org). Animal studies were also approved by the Institutional Animal Care and Use Committee (IACUC) animal experimental Ethics Committee of Taipei Medical University. The animal experiments approval number was LAC‐2019‐0308. Consent to participate is not applicable.

## Consent

The authors have nothing to report.

## Conflicts of Interest

The authors declare no conflict of interest.

## Data Availability

All data analyzed or generated during the study are included in this published article.
